# Exploring the nonlinear piezoresistive effect of 4H-SiC and developing MEMS pressure sensors for extreme environments

**DOI:** 10.1038/s41378-023-00496-1

**Published:** 2023-04-03

**Authors:** Chen Wu, Xudong Fang, Qiang Kang, Ziyan Fang, Junxia Wu, Hongtao He, Dong Zhang, Libo Zhao, Bian Tian, Ryutaro Maeda, Zhuangde Jiang

**Affiliations:** 1 State Key Laboratory for Manufacturing Systems Engineering, International Joint Laboratory for Micro/Nano Manufacturing and Measurement Technologies, Xi’an Jiaotong University, Xi’an, 710049 China; 2 School of Mechanical Engineering, Xi’an Jiaotong University, Xi’an, 710049 China; 3 Shandong Laboratory of Yantai Advanced Materials and Green Manufacturing, Yantai, 265503 China; 4 Xi’an Jiaotong University (Yantai) Research Institute for Intelligent Sensing Technology and System, Xi’an Jiaotong University, Xi’an, China; 5 HeBei Semiconductor Research Institute, Shijiazhuang, 050051 China

**Keywords:** Electrical and electronic engineering, Sensors

## Abstract

Microelectromechanical system (MEMS) pressure sensors based on silicon are widely used and offer the benefits of miniaturization and high precision. However, they cannot easily withstand high temperatures exceeding 150 °C because of intrinsic material limits. Herein, we proposed and executed a systematic and full-process study of SiC-based MEMS pressure sensors that operate stably from −50 to 300 °C. First, to explore the nonlinear piezoresistive effect, the temperature coefficient of resistance (TCR) values of 4H-SiC piezoresistors were obtained from −50 to 500 °C. A conductivity variation model based on scattering theory was established to reveal the nonlinear variation mechanism. Then, a piezoresistive pressure sensor based on 4H-SiC was designed and fabricated. The sensor shows good output sensitivity (3.38 mV/V/MPa), accuracy (0.56% FS) and low temperature coefficient of sensitivity (TCS) (−0.067% FS/°C) in the range of −50 to 300 °C. In addition, the survivability of the sensor chip in extreme environments was demonstrated by its anti-corrosion capability in H_2_SO_4_ and NaOH solutions and its radiation tolerance under 5 W X-rays. Accordingly, the sensor developed in this work has high potential to measure pressure in high-temperature and extreme environments such as are faced in geothermal energy extraction, deep well drilling, aeroengines and gas turbines.

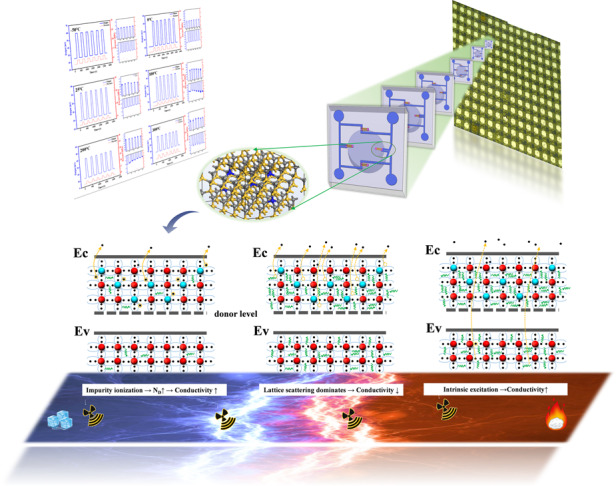

Microelectromechanical system (MEMS) high-temperature pressure sensors are widely used across industrial automation, such as in oil pipelines, water conservancy and hydropower, aerospace, petrochemicals, oil wells, and electric power^1,2^. In addition to high temperatures, these devices are also exposed to electromagnetic radiation, corrosive gas environments, and other harsh factors. Under such working conditions, pressure monitoring during equipment operation is a major problem^3^. Typical MEMS pressure sensors employ silicon (Si). In the temperature range below 100 °C, commercial Si pressure sensors are mature, small, and offer good performance. However, when they are used in environments hotter than 150 °C, current leakage in the internal PN junction directly degrades sensor performance or even causes failure due to the narrow Si band gap^4^. In addition, at high temperature, Si not only undergoes plastic deformation but is also prone to oxidation and corrosion. Hence, traditional Si-based sensors realize pressure measurement at 150 °C by incorporating a heat-dissipation structure or adding a water-cooling jacket. Furthermore, pressure tubes are widely used for conduction measurements. However, these indirect measurement methods lose pressure pulsation and cause signal hysteresis^4,5^. The application of Si MEMS sensors at higher temperatures and in harsh environments is thus limited.

A high-temperature sensor should include a high-temperature material, and the working temperature of a sensor is related to the temperature tolerance of its substrate material. For this reason, the feasibility of preparing high-temperature sensors with high-temperature-resistant materials was studied. As a representative third generation wide-bandgap semiconductor, silicon carbide (SiC) has been used to fabricate insulated gate bipolar transistors (IGBT)^6,7^ and metal-oxide-semiconductor field-effect transistors (MOSFETs)^8–10^, which are mainstream next-generation power devices that offer faster switching speeds and ultrahigh operating voltages. Such excellent performance is predicated on the electrical advantages of SiC, including a large bandgap and high saturated carrier migration rate. It is worth mentioning that SiC also has high thermal conductivity, good radiation resistance, acid and alkali corrosion resistance, and more^11,12^. These mechanical, chemical, and electrical advantages support the inherent advantages of SiC as a substrate for high-temperature MEMS devices^4^.

The application of SiC in MEMS pressure sensors has evolved with single-crystal SiC growth technology. Previously, limited by the epitaxy-based growth of bulk single-crystal SiC, most studies focused on depositing 3C-SiC films on Si-based substrates to form structurally sensitive layers. The fabricated piezoresistive^13–15^ and capacitive^16,17^ 3C-SiC on Si verified the feasibility of SiC as a pressure-sensitive material. However, there is a difference in the thermal expansion coefficient between Si and 3C-SiC, which leads to lattice mismatch at high temperatures, introduces noise and increases the possibility of failure. Therefore, the application of this type of device is greatly limited^4^. With the maturity of 6H-SiC and 4H-SiC single crystal growth technology and the commercialization of epitaxial wafers, a pressure sensor chip with higher temperature tolerance and better performance can be fabricated using all-SiC material as the substrate and has become the subject of mainstream research. From the perspective of pressure sensing, there are mainly three types of SiC pressure sensor chips. Among them, the capacitive type realizes pressure measurement such that the output capacitance value changes due to the change in pole distance after the diaphragm is subjected to a pressure load^18–21^. The advantage of this type is that it is less affected by temperature; however, it suffers from the drawback that the sensor chip is easily disturbed by parasitic capacitance signals. The optical fiber type mainly uses the SiC substrate as structural support^22–24^ and the optical fiber as the pressure sensor. This method can realize pressure measurements at higher temperatures, but the modulation and demodulation of the optical signal is complicated. Therefore, the third type, the piezoresistive type, is the most commonly used and has the advantages of a simple structure and convenient output signal acquisition^25,26^. The earliest related research was conducted by RS. Okojie et al. ^27,28^. They developed a piezoresistive pressure-sensitive chip using a 6H-SiC substrate. Subsequently, they focused on improving the working temperature of the chip and conducted a series of related studies on chip electrodes. In recent years, Dzung Viet Dao et al. ^29–31^ have been working on the application of SiC in MEMS sensors and have continued to fabricate and test piezoresistive pressure sensor chips based on P-type 4H-SiC. In summary, it is clear that the emergence of SiC and its application in high-temperature sensor devices may provide a better choice for sensor chip substrates. However, difficulties and challenges remain in this field. First, there is little research on the structural optimization design of the SiC sensor chip; specifically, there is little research on the piezoresistive effect of SiC, which results in a lower sensitivity of the sensor. Second, due to its poor high-temperature stability, there is no output repeatability testing of the SiC piezoresistive sensor, which limits the practical engineering applications of these SiC pressure sensors at high temperatures.

In this study, an NPN-type electrically isolated 4H-SiC piezoresistive pressure sensor chip was proposed. First, the nonlinear piezoresistive effect of 4H-SiC over a wide temperature range was explored. Piezoresistor samples were prepared using the same substrate as the fabricated sensor chip. The cantilever beam bending method was used to study the piezoresistive effect of N-type 4H-SiC, and the transverse piezoresistive gauge factor (GF_t_) and longitudinal piezoresistive gauge factor (GF_l_) of the 4H-SiC were measured. In addition, nonlinear variation of the piezoresistors from −50 to 500 °C was investigated, and a model for conductivity variation based on scattering theory was developed to reveal the underlying mechanism. Then, the optimal dimension parameters of the sensor chip were obtained by combining the theoretical calculations with a simulation optimization algorithm. Finally, repeatable output test results of the packaged pressure sensor from −50 to 300 °C show high output sensitivity and low temperature coefficient sensitivity (TCS). Moreover, experiments verified the survival performance of the SiC pressure sensor in harsh environments, including the ability to withstand acidic and corrosive environments and electromagnetic radiation from X-rays.

## Results and discussion

### The piezoresistive effect of N-type 4H-SiC

Because the piezoresistive structure is the optimal choice for fabricating 4H-SiC high-temperature pressure sensor chips^32^, to design MEMS pressure sensor chips with SiC bulk material, the piezoresistive effect of 4H-SiC should be investigated. According to the piezoresistive effect of semiconductor SiC^31,33,34^, the change in resistance of a semiconductor after deformation by pressure load is mainly due to the change in resistivity, which has a positive correlation with deformation strain. The degree of strain-induced change in resistivity directly determines the output sensitivity of the sensor, and we define this correlation in terms of the piezoresistive gauge factor GF^35^. To ensure that the piezoresistive test results support chip design and fabrication with the same substrate parameters, it is necessary to investigate the GF value of N-type 4H-SiC with a doping concentration of 1e19 cm^−3^ before sensor chip design. On the other hand, the dimensions of the piezoresistor also impact the GF value for a certain doping concentration^36–39^.

**Fig. 1:**
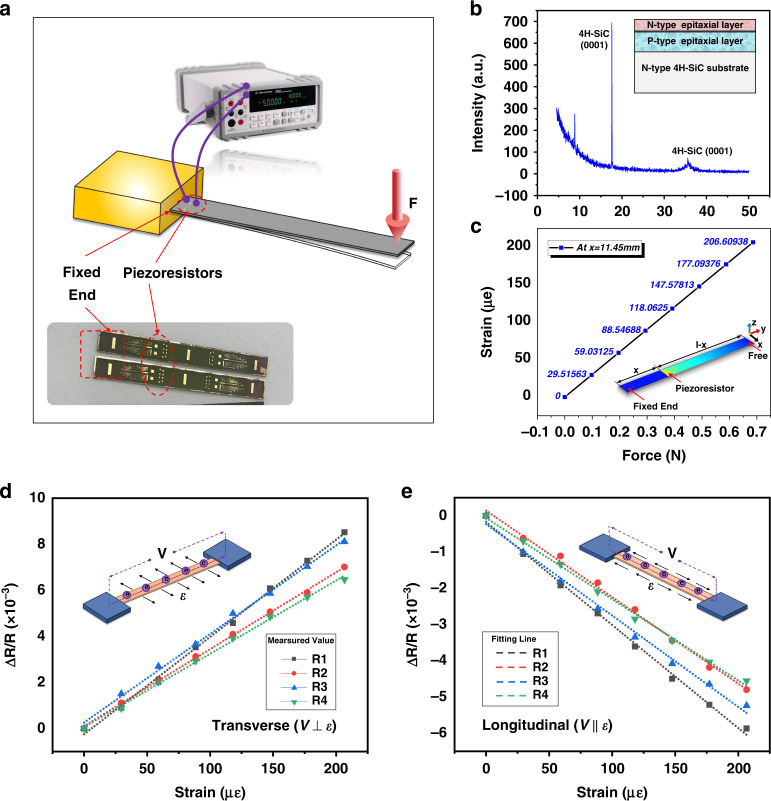
Piezoresistive effect of N-type 4H-SiC. **a** Schematic diagram of the experimental setup for piezoresistive effect testing. **b** 4H-SiC epitaxial wafer parameters and X-ray diffraction characteristic peaks. **c** Surface strain distribution of piezoresistive test samples under F load. **d** Fitted curve of the transverse piezoresistive coefficient GF_l_. **e** Fitting curve of the longitudinal piezoresistive factor GF_t_

To study the piezoresistive effect of N-type 4H-SiC, equal-section cantilever beam bending was applied^40^ as illustrated in Fig. 1a, where we fixed one end of the sample and applied a specific force F to the other end. The cantilever beam bending generated a strain at the location where the piezoresistors were located, and the resistance value change was measured, which resulted in a difference in the value of GF^35^. A schematic of the doped epitaxial structure of the wafer used to study the piezoresistive effect is shown in Fig. 1b. The surface n-type epitaxial layer was heavily doped with a concentration of 1e19 cm^−^^3^ and a thickness of 2 μm. The piezoresistive test sample was formed after a series of micro- and nano-processes, such as surface shallow etching, SiO_2_ insulating layer deposition, and metal ohmic contact fabrication. The applied force range was 0–0.686 N and the piezoresistors on the surface of the test sample generated a corresponding strain *ε* .We designed four combinations of length (*L*) and width (*W*) of piezoresistors (L400 × W10, L200 × W30, L400 × W20, and L200 × W10) to investigate the piezoresistive effect of 4H-SiC and ensure the maximum sensitivity of the sensor at the design stage. As shown in Fig. 1a, the total size of the piezoresistor sample was 33 mm × 5 mm × 0.35 mm and the center of the piezoresistors was 11.45 mm from the fixed end of the sample. To further calculate the piezoresistive coefficient GF, the strain value of the piezoresistors on the cantilever beam needs to be determined by simulation. Therefore, a cantilever piezoresistive model was established using the multiphysics field coupling simulation software COMSOL. The sample size and piezoresistor distribution are consistent with the experimental sample, as shown in the embedded rectangular cantilever structure in Fig. 1c. In simulation, the same loads and constraints were imposed on the cantilever as in the experiment (one end was fixed, and a force F was applied at the other end). Considering the linear distribution of strain values on the piezoresistors, we consider the strain value at the geometric center point of the piezoresistor as the final strain value. It is worth noting that this value is also equal to the average strain of the whole piezoresistor. Finally, the strain induced in the piezoresistors was extracted and then plotted in Fig. 1c. The longitudinal piezoresistive coefficient GF_l_ is used to describe the change in resistance of the piezoresistor when the voltage *V* and strain *ε* are parallel. In contrast, the transverse piezoresistive coefficient GF_t_ is used to describe the piezoresistive effect when the directions of *V* and *ε* are perpendicular. The research and calculation of GF_l_ and GF_t_ are crucial to sensor chip design and are required for designing SiC piezoresistive pressure sensors.

Figure 1d, e shows the fitted curve of the GF value and Table 1 lists the final GF values of the piezoresistors with four different dimensions. It can be concluded that the original resistance value of a piezoresistor with different length and width is related to the geometry of the piezoresistor. The sheet resistances of the piezoresistors were the same and equal to approximately 100 Ω/□. Due to the nonuniformity in doping of the piezoresistors and error in lithography manufacturing, there is a controllable error between the theoretical design and actual measured resistance values. For the n-type 4H-SiC, the values of GF_t_ and GF_l_ showed opposite trends with respect to strain. The resistance value of transverse piezoresistors increases with increasing strain, whereas that of longitudinal piezoresistors decreases with increasing strain. The absolute value of GF_t_ is always greater than that of GF_l_ regardless of the length and width of the piezoresistors. This reveals new findings on the n-type 4H-SiC piezoresistive effect, which is a meaningful basis for further sensor chip design. In comparison, the piezoresistor of size L400 × W10 has greater GF_l_ and GF_t_. Therefore, based on the above research, the dimensions of the piezoresistor sensor were determined.

**Table 1:** The GF values corresponding to different piezoresistor dimensions

Name	Dimension (μm)	Sheet resistance (Ω/□)	Theoretical resistance value (kΩ)	Measured resistance value (kΩ)	Transverse GF_t_	Longitudinal GF_l_
R1	L400 × W10	100	4	3.25	40	−26.5
R2	L200 × W30		0.667	0.533	33.4	−23.9
R3	L400 × W20		2	1.902	35.4	−24.3
R4	L200 × W10		2	1.825	31.8	−22.4

### Structure of the sensor chip and microscopic details

**Fig. 2:**
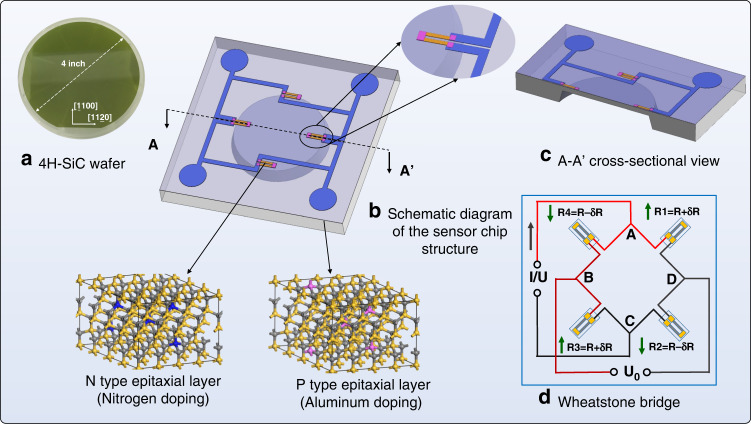
Basic conceptual design of the sensor chip. **a** Green transparent 4H-SiC wafer. **b** Schematic of the sensor chip structure. **c** A-A’ cross-sectional view. **d** Wheatstone bridge connection

After obtaining the 4H-SiC piezoresistor dimensions with the largest piezoresistive effect, we further design the structure of the piezoresistive 4H-SiC pressure sensor chip. An NPN-type electrically isolated piezoresistive pressure sensor chip was developed in this study using the third-generation wide bandgap semiconductor single crystal 4H-SiC as the substrate. Figure 2a shows the 4H-SiC substrate wafer used in the sensor chip fabrication, which was purchased from Dongguan Tianyu Co., Ltd., China. Unlike Si wafers, which appear opaque gray–black after ingot slicing, single-crystal 4H-SiC wafers are translucent green after ingot slicing, and this is also a trait that separates them from Si wafers. Additionally, multilayer epitaxial structures were designed to achieve electrical isolation. The doping concentration of the surface epitaxial n-type strongly doped layer was 1e19 cm^−3^, while the thickness of the n-type substrate was 350 μm. There was a p-type epitaxial layer between the substrate and the n-type layer with a doping concentration of 5e18 cm^−^^3^. The thicknesses of the p-type and n-type epitaxial layers were 5 and 2 μm, respectively. The developed 4H-SiC piezoresistive circular diaphragm pressure sensor chip is shown schematically in Fig. 2b. The epitaxial layers were obtained by doping growth on the single crystal substrate. The sensitive piezoresistors were formed on the surface highly doped n-type epitaxial layer, and the PN junction isolation was formed by an intermediate p-type doped layer. The structure contains a deformable sensitive diaphragm thanks to the high-quality etching effect of the plasma dry etching method. Square and circular shapes are the most commonly used shapes in Si-based structures. Considering the high hardness and anti-corrosion properties of SiC^3^, when compared with a square diaphragm, the circular diaphragm has the advantages of uniform force distribution and low stress concentration, making it an ideal diaphragm shape for brittle semiconductor substrates such as 4H-SiC. Four sets of piezoresistors were arranged symmetrically around the edge of the circular diaphragm, as shown in Fig. 2b. When the diaphragm is under a pressure load, the strain of the piezoresistor varies, resulting in a change in the resistance value. The electrical connection between the piezoresistors is achieved via a Wheatstone bridge, as shown in Fig. 2d.

**Fig. 3:**
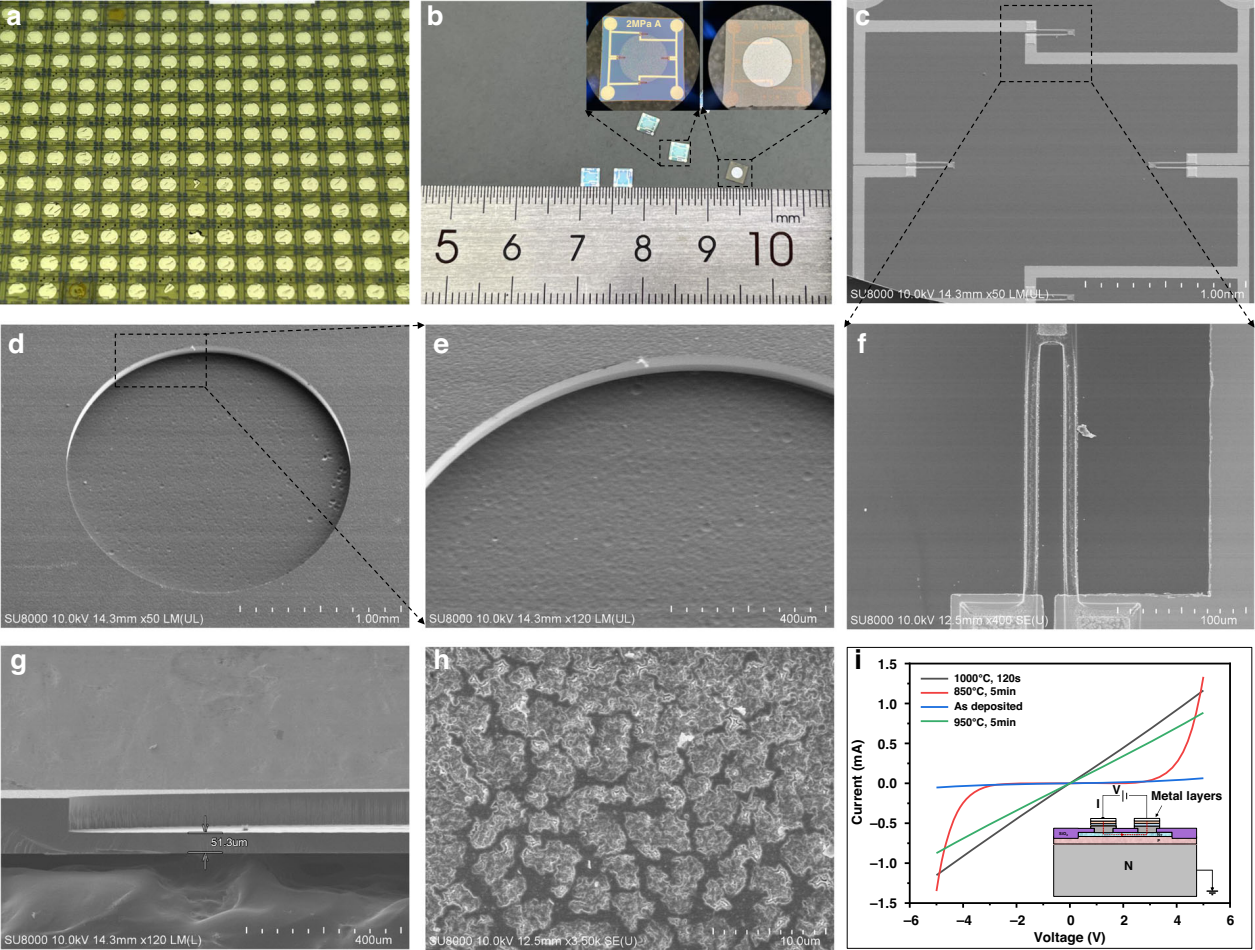
**a** The fabricated SiC piezoresistive pressure sensor chip within one 4-inch SiC wafer. **b** The SiC piezoresistive pressure sensor chip unit and overall dimensions. **c** SEM microstructure of the piezoresistor arrangement on the chip. **d**, **e** SEM microstructure of the sensitive diaphragm back cavity. **f** Enlarged SEM microstructure of piezoresistors. **g** SEM of the cross-sectional view of the chip (sensitive diaphragm thickness can be observed and measured). **h** SEM microstructure of the metal layer in the ohmic contact area. **i** Metal/SiC ohmic contact electrical equivalent model and *I*–*V* characteristics after annealing at different temperatures

Figure 3 shows details of the fabricated chip. Hundreds of sensor chips were fabricated on a 4-inch 4H-SiC wafer, with individual chip dimensions 3 mm × 3 mm × 180 μm and a sensitive diaphragm thickness of 51 μm, which was consistent with the design parameters. The surface-sensitive piezoresistors were symmetrically distributed. The back cavity of the sensor chip had high flatness, which ensured sensor output linearity and hysteresis. The electrical model between the piezoresistors is shown in the inset of Fig. 3i, and the measured current-voltage characteristics are obtained from ohmic contacts.

### Temperature effect of 4H-SiC piezoresistors

The change in semiconductor resistance with temperature is considered as the resistance temperature effect of a material, and the temperature coefficient of resistance (TCR) is used to quantitatively evaluate the significance of this effect^41^. Herein, the relationship between the variation in resistance values of the SiC piezoresistor and temperature was determined by measuring the resistance value of n-type heavily doped 4H-SiC at high temperatures.

**Fig. 4:**
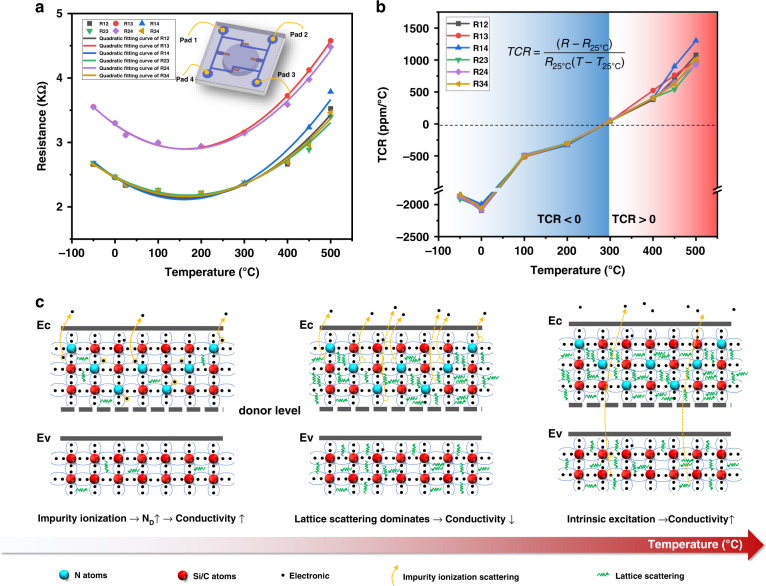
Temperature effect of 4H-SiC piezoresistors. **a** The change in the piezoresistor resistance value of the four metal pads on the sensor chip at different temperatures. **b** The resistance temperature coefficient (TCR). **c** The microscopic motion mechanism of carriers at different temperatures. At a certain temperature interval (low temperature), the carriers are generated by the ionization of doped impurities, and as the temperature increases, the carrier concentration increases, the impurity scattering effect is weakened, and the conductivity increases. When the temperature increases (middle temperature), the impurity ionization reaches saturation, at which time the carrier concentration no longer changes, lattice vibration scattering becomes the main factor affecting the carrier mobility, and the strong lattice vibration decreases the carrier mobility, leading to a decrease in conductivity. With a further increase in temperature (high temperature), the lattice vibration continues to act, but the intrinsic excitation will make the carrier concentration rise rapidly, and the effect is more significant. The comprehensive effect is reflected in the increase in conductivity, which is also close to the limit of the semiconductor device temperature

When applying the resistance temperature effect to the fabrication of devices, such as thermal sensors, it is desirable to achieve a higher temperature resolution by changing the resistance value as much as possible when the temperature changes. However, in the case of SiC piezoresistive high-temperature pressure sensors, this change in resistance due to temperature is not constant, which causes temperature drift in the output voltage that needs to be avoided and minimized to ensure sensor performance over a wide temperature range. To investigate the effect of temperature on the chip piezoresistors, the change in chip resistance values for different temperatures is shown in Fig. 4. As shown in the embedded figure, the chip’s metal pads are numbered from 1 to 4 and there are six resistance values between these metal pads across the closed-loop Wheatstone bridge, namely, R12, R13, R14, R23, R24, and R34. All six resistance values were recorded to monitor the change in resistance accurately. When the temperature was lower than room temperature, the resistance increased gradually as the temperature decreased. However, when the temperature was higher than room temperature, the change in resistance was not monotonic. The resistance value first decreased while the temperature was between 100 and 200 °C, after which the resistance value continued to increase. Furthermore, a quadratic polynomial fit was used to determine the critical temperature inflection points for the increasing and decreasing resistance values. For six sets of resistance curves, the temperature inflection points for resistance rise and fall were obtained as 200, 225, 245, 160, 220, and 185 °C. After removing the maximum and minimum temperatures, the mean value of the temperature inflection point was 207.5 °C. The nonlinear change in resistance reflected the microscopic changes within the doped semiconductor piezoresistance at high temperature, which may lead to a change in the sensor output sensitivity. For sensor performance, the resistance temperature coefficient (TCR) is defined as

1 $$TCR = (R - R_{25^ \circ {{{\mathrm{C}}}}})/[R_{25^ \circ {{{\mathrm{C}}}}}(T - T_{25^ \circ {{{\mathrm{C}}}}})]$$

TCR not only reflects the trend and variation of resistance with temperature but can also be used to quantitatively evaluate the dependence of resistance on temperature drift. According to the series and parallel relationship of the Wheatstone bridge, the resistance values R13 and R24 are theoretically equal to the piezoresistor resistance of a single bridge arm. Table 2 calculates the TCR values of the bridge arm resistance values R13 at different temperatures. The experimental results in Fig. 4b illustrate that a negative TCR appeared at temperatures below 300 °C. The TCR value reaches a negative maximum (−2092 ppm/°C) near 0 °C. This means that at this temperature point, the resistance value is fully influenced by the temperature and changes rapidly. At 300 °C, the positive TCR was as low as 41.324 ppm/°C, followed by positive TCR values at high temperatures. In the temperature range of 100–450 °C, the TCR value, although experiencing a change from negative to positive values, showed a better linearity overall, and the difference in TCR values between different resistances was small. At 450 °C, the maximum value of TCR was only 899.33 ppm/°C, indicating that below 450 °C, the resistance changes were more stable and regular, which implied a more stable sensor chip output performance. When the temperature exceeded 450 °C, the TCR value began to show significant variability, and at 500 °C, the TCR exceeded 1000 ppm/°C, indicating that the output of the sensor may fluctuate significantly. In fact, the TCR value is not only affected by temperature but is also related to the doping concentration and type of the SiC piezoresistive layer. For undoped and lightly doped (less than 1e18 cm^−3^) 4H-SiC, the TCR values were up to 7600 ppm/°C^34^, and the higher the doping concentration, the weaker the temperature dependence of the resistance values was, thus exhibiting smaller TCR values. In the results of Alexander A. Ned^42^, for 6H-SiC with a doping concentration of 2e19 cm^−^^3^, the values of TCR corresponding to n-type and p-type resistances at 100 °C were −2400 and −7400 ppm/°C, respectively, and there was a significant decrease in the value of TCR as the temperature increased to 250 °C (although it was still negative). Okejie et al. demonstrated^43^ that the output sensitivity of piezoresistive SiC pressure sensors showed a trough near 300 °C, followed by a tendency for the sensitivity to rebound as the temperature increased. This interesting phenomenon was possibly because of the thermal stress deformation caused by encapsulation, and further investigations are needed.

**Table 2:** The TCR of R13 at different temperatures

Temperature(°C)	−50	0	25	100	200	300	400	450	500
TCR (ppm/°C)	−1874.6	−2087.0	0	−490.7	−310.2	60.5	407.3	653.3	925.4

By measuring the resistance value of n-type doped 4H-SiC at high temperature, the changing relationship between the resistance and temperature was found. As the temperature increased, the resistance first decreased until the temperature reached 207.5 °C, after which it gradually increased back to the original resistance at room temperature. When the temperature exceeded 300 °C, the resistance was greater than the original value and continued to increase. This fundamentally explains the change in the sensitivity output of the piezoresistive SiC pressure sensor, which may be closely related to the change in the resistance of the piezoresistor. Furthermore, the internal factors of this nonlinear change in highly doped n-type 4H-SiC with temperature change are worth studying.

According to semiconductor physics theory^44^, the conductivity *σ* of a doped impurity semiconductor satisfies the relationship $\sigma \,\,{{{\mathrm{ = }}}}\,\,nq\mu _n + nq\mu _p$, where *n* is the carrier concentration and *μ* is the carrier mobility. This relationship implies that the conductivity of a semiconductor depends on the concentration distribution of carriers and their magnitude of mobility. At a constant temperature, ideal carriers are continuously accelerated by an external electric field. In fact, these carriers continuously collide with the vibrating lattice atoms or impurity ions in the process of free motion, changing the original velocity magnitude and direction, and thus, the periodic potential field is disrupted. Such collisions are attributed to scattering effects that include mechanisms such as lattice vibrational scattering, ionized impurity scattering, and intervalley scattering, and it is seen that these scattering effects hinder carrier motion. The microscopic motion mechanisms of the carriers at different temperatures are shown in Fig. 4c. At a certain temperature interval (e.g., low temperature), carriers are generated by the ionization of doped impurities, and as the temperature increases, the carrier concentration increases, the impurity scattering effect is weakened, and the conductivity increases. When the temperature increases (middle temperature), the impurity ionization saturates, at which time the carrier concentration no longer changes. Lattice vibration scattering becomes the main factor affecting carrier mobility, with a strong lattice vibration decreasing the carrier mobility and leading to a decrease in conductivity. With a further increase in temperature (high temperature), the lattice vibration continues to act, but the intrinsic excitation causes the carrier concentration to rise rapidly and this effect becomes more significant. This phenomenon is reflected in the increase in conductivity, which is also close to the limit of the semiconductor device at this temperature range.

From the above analysis, it can be determined that the change in conductivity with temperature is a complex interaction process influenced by carrier concentration and mobility. Because resistivity and conductivity have a reciprocal relationship, this model explains the experimental phenomenon in which conductivity first drops and then rises. The above model clarified the mechanism of 4H-SiC piezoresistance change with temperature and created a theoretical basis for further utilizing the piezoresistive effect to develop various sensing devices. In addition, through the comparison of TCR values^34,42,43^, according to the test results, the resistance value of the piezoresistors is stable and fluctuates within a small range below 450 °C, and the 4H-SiC piezoresistors have small TCR values. These two significant advantages imply that the piezoresistive effect of 4H-SiC is less affected by high temperatures. Therefore, this comprehensive and systematic research on the piezoresistive properties of 4H-SiC plays an important role in subsequent 4H-SiC piezoresistive pressure sensor design.

### Output characteristics of the pressure sensor chip

The traditional pressure sensor performance measurement is carried out by means of a static single-point pressure test. This includes a lack of features that reflect real-time sensor feedback on pressure. Additionally, for high-temperature pressure measurements over 150 °C, there are few real-time, multiple-repetitive pressure test results. Therefore, it is currently difficult to search for a standard for evaluating the static performance of pressure sensors at high temperatures. Hence, in this work, real-time repeatability measurement of SiC high-temperature pressure sensors over a wide temperature range is reported for the first time. First, this result can prove the repeatability of SiC sensors at high temperatures. Second, it implies that the sensor has stable working characteristics, not just in the laboratory prototype test stage, but when directly applied to actual engineering measurement. We studied the hydrostatic output of the sensor chip at six key temperature points between −50 and 300 °C (−50, 0, 25, 100, 200, and 300 °C), holding the sensor for 1.5 h at each temperature point and waiting for the output voltage to stabilize before testing. It is worth noting that the data acquisition system was embedded in a control PC to conduct a complete repeatability test for the sensor at high temperatures. The output characteristics, such as hysteresis and linearity at high temperatures, are calculated to demonstrate the valuable engineering applications of this sensor (despite having been rarely presented in previous studies).

**Fig. 5:**
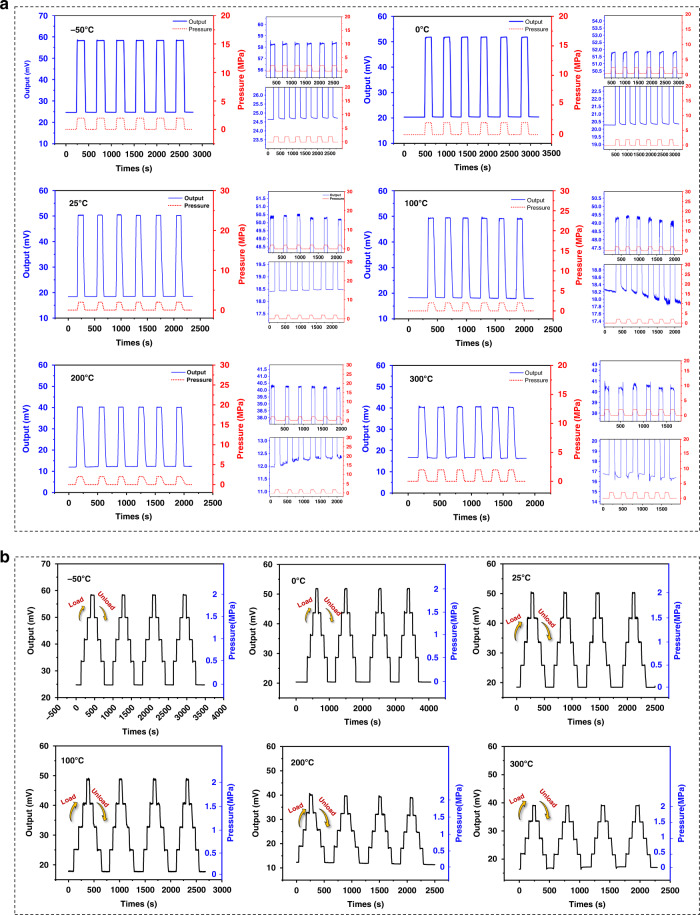
Output characteristics and high-temperature repeatability testing. **a** Full-scale output. (For the cyclic measurement curve at each temperature point, an enlarged figure of the full-scale steady-state output when the pressure reaches 2 MPa is given in the upper right corner. Similarly, an enlarged picture of the steady-state output after pressure unloading is shown in the lower right corner. This is done so that subtle changes in the output can be observed.) **b** Sensor output of forward loading and reverse unloading

Figure 5a demonstrates the full-scale output (FSO) characteristics of the sensor. The sensor was held for 2 min under a full-scale load and kept at zero load for 3 min after unloading. On observing a macroscopic view of the output curve, the sensor FSO at room temperature was approximately 31.8 mV and the sensitivity was calculated to be 3.18 mV/V/MPa. In addition to the FSO characteristics, Fig. 5b shows the test results of the sensor chip during gradual pressure addition in the forward loading and reverse unloading strokes, with a maintenance pressure loading time of 50 s at each load. The data reflect the repeatability and hysteresis characteristics of the sensor. Table 3 lists the calculated main performance characteristics of the sensor at room temperature (25 °C), low temperature (−50 °C) and high temperature (300 °C), and the sensor accuracy at room temperature reaches 0.56% FS. The performance degradation at low temperature is better than that at high temperature. Overall, the sensor has a small hysteresis.

**Table 3:** Output characteristics of the sensor chip

Temperature (°C)	Sensitivity (mV/V/MPa)	TCS (FS/°C)	TCO (FS/°C)	Linearity (%FS)	Hysteresis (%FS)	Accuracy (%FS)
−50	3.38	−0.073%	−0.45%	0.77	0.14	1.03
25	3.18	/	/	0.1	0.28	0.56
300	2.4	−0.067%	−0.037%	1.94	0.77	2.18

**Fig. 6:**
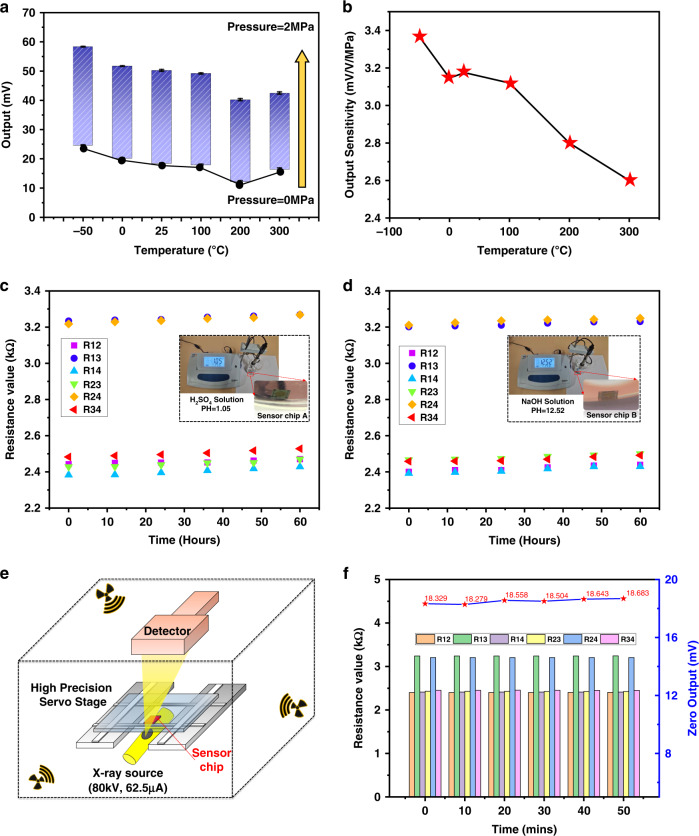
**a** Full-scale output histogram and zero-pressure output change curve at different temperatures. **b** Sensitivity versus temperature curve. **c**, **d** The resistance value of two chips when immersed in H_2_SO_4_ (pH = 1.05) and NaOH (pH = 12.52) solutions, respectively. **e** Schematic diagram of the sensor being irradiated by industrial CT. **f** The resistance value and zero output change after X-ray irradiation

The repeatability and stability of the sensor at various temperature points were good, and after reaching a high temperature of 300 °C, there was a small drift in the output of the chip at steady state. We also provide a detailed plot of the output curve in the full-scale and zero-pressure states, which shows that the overall output fluctuation is small. At the instant of pressure loading and unloading, the curve has a burr at the inflection point of the pressure change due to the manual operation of the pressure controller. This burr is caused by manual operation error and reflects the sensitive response of the sensor to the pressure load. The output results show that the full-scale output FSO of the sensor decreased as the temperature increased. At −50 °C, the FSO increased by 2 mV compared to room temperature, and the corresponding sensitivity increased to 3.38 mV/V/MPa. Conversely, at 300 °C, the FSO decreased by 7.8 mV, at which time the sensitivity was 2.4 mV/V/MPa. Figure 6b shows the sensitivity change of the sensor chip at different temperature points, and the sensitivity temperature coefficient (TCS) of the sensor at low temperature (−50 °C) and high temperature (300 °C) can be calculated with TCS values of −0.073% FS/°C and −0.067% FS/°C, respectively. The small TCS of the sensor compared to Si-based pressure sensors^45,46^ as well as other reported sensor performances on SiC^34,42^ indicates its excellent high-temperature stability. For high-temperature sensors, the zero position of the sensor drifts as the temperature changes, which is an important indicator for evaluating high-temperature stability. Figure 6a shows the histogram of the zero drift of the sensor at different temperatures. The zero drift of the sensor at −50 °C and 300 °C are −0.45% FS/°C and −0.037% FS/°C, respectively. The small zero drift of the sensor at high temperatures also implies its potential application in the high-temperature field.

It is worth noting that the theoretical value of the FSO of the designed sensor by simulation is 32.45 mV, which is slightly larger than the measured value (31.8 mV). Because the anisotropic Young’s elastic modulus of 4H-SiC was considered in the structural optimization design, the theoretical design value and the measured value are quite close. However, the process error in the sensor chip manufacturing process affect the final test performance of the sensor chip. For the 4H-SiC piezoresistive pressure sensor chip designed in this work, one of the important processes is diaphragm etching. Since 4H-SiC has a large elastic modulus and does not chemically react with any acid or alkali at room temperature, it is a hard and brittle single crystal material that is difficult to process^2^. Therefore, in the 4H-SiC dry etching process, the etching thickness of the diaphragm should be as close as possible to the designed thickness. However, the control of the actual etch rate depends on the precision of the etching machine (including the gas flow rate and power). To ensure reliability of the 4H-SiC diaphragm in subsequent repeated tests under pressure load, we were generally more conservative, such that the diaphragm thickness after etching is slightly greater than the design value (rather than risk over-etching). In this work, the measured diaphragm thickness (approximately 51.5 μm) is slightly larger than the design value (51 μm), and this error is highly likely to be the main reason for the deviation between the test and simulation results. Therefore, higher precision process technology will promote even better performance of sensors.

Under harsh working conditions, in addition to high-temperature challenges, there are also acidic or alkaline gas atmospheres, which react with water to produce acidic or alkaline harsh environments. To evaluate the ability of the sensor to withstand acidic- and alkaline-exposed harsh environments, the sensor chip was submerged in a H_2_SO_4_ solution with a pH value of 1.05 and a NaOH solution with a pH value of 12.52 for 60 h, and the resistance values were measured every 12 h. Consistent with the resistance nomenclature in Fig. 4a, the six resistance values between the four metal pads were recorded in Fig. 6c. The resistance values show small incremental changes with increasing immersion time; when the 60th hour was reached, the resistance values increased by 1.398% in the H_2_SO_4_ solution and 0.343% in the NaOH solution, which verifies that the chips have high tolerance to corrosive environments. In addition, to investigate the effect of radiation on the sensor performance, the sensor was irradiated in an industrial CT chamber, as shown in Fig. 6e. The X-ray source voltage was 80 kV, the current was 62.5 μA, and the irradiation power was 5 W. A total of five irradiations were conducted, each for 10 min, and the resistance value of the sensor chip was measured after irradiation as well as at the zero output, as shown in Fig. 6f. The maximum fluctuation of the sensor resistance value is 0.125%, and the maximum change of the sensor zero output is 1.93%. X-ray radiation is widely present in harsh industrial environments, and an electromagnetic wave with very short wavelength and high energy can easily interfere with sensor devices, distorting monitoring signals. The above experimental results verify that our piezoresistive pressure sensor with the SiC substrate can not only measure pressure in a high-temperature environment but also withstand a corrosive environment and resist the influence of electromagnetic radiation.

Table 4 summarizes the comparison between this study and other publicly reported results. Multiple repetitive loading tests of the SiC pressure sensor at high temperatures were performed for the first time, which led to broader performance indicators and is of far-reaching significance for practical engineering applications. Moreover, the sensitivity of the sensor is high because the piezoresistive effect of n-type 4H-SiC has been thoroughly studied and detailed sensor optimization has been carried out. In fact, the piezoresistor temperature characterization study shown in Fig. 4 demonstrates that the sensor chip can operate at 500 °C in air. However, as limited by the high-temperature performance of the sensor package, effective repeatability tests in this study were conducted up to 300 °C. In the future, in addition to further improving the sensor performance, a high-temperature package will be designed to achieve higher temperature repeatability testing and performance verification.

**Table 4:** Performance summary and comparison of different sensors

References	Piezoresistors doping type	Maximum output test temperature (°C)	Output Repeat test (Yes or No)	Sensitivity (mV/V/MPa)	Pressure range (MPa)
^53^	N type 4H-SiC	150	No	8.59 mV/mA/MPa	7
^54^	N type 4H-SiC	25	No	2.68	6
^41^	N type 6H-SiC	400	No	0.4	12
^55^	P type 4H-SiC	25	No	10.9	1
^56^	N type 4H-SiC	250	No	1.56	5
This work	N type 4H-SiC	300	Yes	3.38	2

## Methods

### Anisotropic numerical calculation for chip dimension

**Fig. 7:**
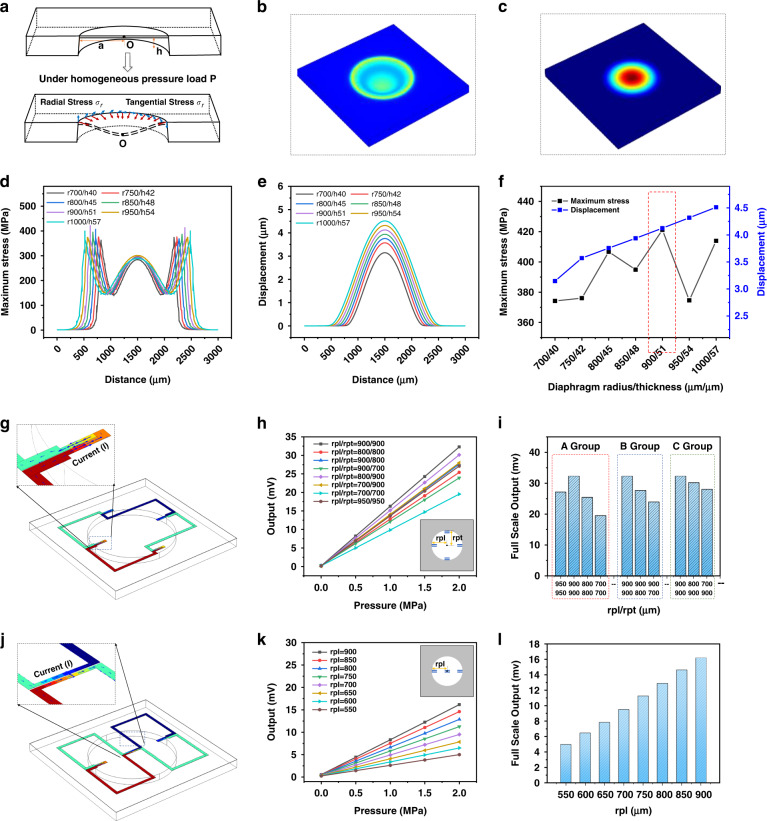
Anisotropic numerical calculation for chip dimension. **a** Deformation of the circular diaphragm under pressure load and the stress at the edge of the diaphragm (including radial stress A and tangential stress B). **b**, **c** Surface stress and displacement distribution of the circular SiC-sensitive diaphragm. **d**, **e** Values of stress and displacement at different positions on the surface of circular SiC diaphragms under different sizes. **f** Comprehensive optimization of the radius and thickness of the sensitive diaphragm. **g**, **j** Potential distribution and current vector diagrams corresponding to two piezoresistor arrangements (circumferential arrangement and radial arrangement). **h**, **i**, **k**, **l** Simulation results of the sensor’s full-scale output when the piezoresistors are located at different positions on the diaphragm

To accurately design the critical dimensions of the piezoresistive SiC pressure sensor chip, we consider the deformation-sensitive diaphragm part as a thin clamped plate, as illustrated in Fig. 7a. According to the Tymosinko thin plate theory^47^, two major principles of sensor-size design can be obtained. First, the diaphragm is bent and deformed by a homogeneous pressure load, and the maximum deformation should satisfy the small deflection deformation category, which means that the maximum bending displacement on the diaphragm should be less than 5% of the total diaphragm thickness. Second, bending and strain lead to different distributions of stresses on the diaphragm surface, and the maximum stress should be guaranteed to be less than 95% of the 4H-SiC material destruction stress limit. The above theoretical design model is derived and calculated based on the parameters of the isotropic elastic properties of 4H-SiC and the key dimensional parameters of the sensor chip, such as the diaphragm thickness and diameter, which can be initially calculated for different pressure load ranges. The related computational theory has been elaborated in our previous work^47^. Crystallographic analysis shows that 4H-SiC belongs to a hexagonal crystal system with a space point group of P6/3m, and the stress–strain relationship of its crystal along different directions is expressed as a 6 × 6 symmetric matrix equation^47,48^. In a 4H-SiC chip substrate deformed under pressure, the material elastic properties were anisotropic. Therefore, the performance parameters of the sensor designed according to the theoretical calculation will have errors when compared with those obtained from experimental tests, and the detailed dimensions of the sensor need to be further optimized by precisely considering the anisotropic response of the material.

Deformation of the diaphragm under pressure is particularly important for sensitivity design of the sensor. When the substrate material is determined, the main parameters affecting the diaphragm deformation are the diaphragm radius a and thickness h. Thus, to obtain a higher sensitivity, the diaphragm radius can be increased, and the diaphragm thickness can be reduced. However, in actual sensor chip fabrication, considering the utilization rate of the original SiC wafer and the size limitation of the sensor package and test space, it is considered that the higher the utilization rate of the wafer is, the better and smaller the overall size of the sensor. Hence, the diaphragm dimension cannot be unlimitedly large. As a result, to approach real deformation more accurately, an equivalent structural optimization model of the 4H-SiC piezoresistive pressure sensor chip based on the anisotropic material properties is established using the software COMSOL, as shown in Fig. 7. Through numerical calculations, the maximum stress and displacement on the diaphragm under different pressure loads can be obtained. Figure 7d shows the stress distribution of the chip diaphragm after compression and deformation, and the results illustrate that for circular diaphragms, the maximum stress occurs at the edges of the diaphragm. The larger the radius of the diaphragm and the thinner its thickness, the higher the stress–strain on the diaphragm is. Figure 7e shows the calculated results of the deformation displacement of the diaphragm, and it can be observed that the deformation displacement is the largest at the center of the diaphragm. Figure 7f illustrates the composite optimization results for the maximum stress and displacement on the membrane. For the designed 2 MPa chip, when the chip diaphragm radius a is 900 μm and the thickness h is 51 μm, the stress on the diaphragm reaches its maximum and the diaphragm deformation does not exceed 5% of the total thickness, which not only achieves the highest chip sensitivity but also ensures that the diaphragm is always in the linear deformation stage. This is an optimized-dimension design for a 2 MPa SiC pressure chip.

In addition to the dimensions of the diaphragm, the piezoresistive arrangement scheme affects the sensitivity of the sensor^47^. Based on the results of the above study on the piezoresistive effect of 4H-SiC, the effects of different piezoresistive arrangement positions on the output sensitivity of the sensor were investigated, and an optimized piezoresistive arrangement scheme was obtained. According to the diaphragm stress distribution in Fig. 7d, the stress around the edge of the circular diaphragm is the highest, and the stress near the center of the circular diaphragm is greater than that in the other locations. Based on this, the optimization calculation for the piezoresistive arrangement scheme includes two steps. The first step is the design of a piezoresistive arrangement scheme, which is mainly reflected in Fig. 7j and g. The second step is determining the optimal distance between the piezoresistors and diaphragm center in each arrangement scheme, which is mainly reflected in Fig. 7h, i and k, l. Figure 7j illustrates the scheme of the piezoresistive surrounding arrangement with four groups of piezoresistors symmetrically arranged at the edge of the circular diaphragm. When under strain, two groups of radially arranged piezoresistors are subjected to strain in the radial direction of the circular diaphragm. When an electric field is applied, the current direction becomes parallel to the strain direction. This case mainly utilizes the longitudinal piezoresistive effect of 4H-SiC, and the other two groups of tangentially arranged piezoresistors were subjected to a strain direction along the radial direction of the diaphragm; therefore, the current direction is perpendicular to the strain direction, which utilizes the transverse piezoresistive effect of 4H-SiC. A pressure load of 2 MPa was applied to the sensor chip, and the chip output comparison results in Fig. 7h, k show that this arrangement ensures higher sensitivity when the piezoresistors are arranged around other piezoresistors compared with the radial arrangement of the piezoresistors because the transverse piezoresistive effect of 4H-SiC is better utilized in the former case. The calculated results in Fig. 7h indicate that the piezoresistor position has a significant effect on the chip output, and a detailed investigation is needed to better explore this phenomenon.

The comparison of the three groups in Fig. 7i demonstrates that the change in position of the piezoresistors using the transverse piezoresistive effect has a significant effect on the output of the sensor when using the longitudinal piezoresistive effect, which is consistent with the conclusions obtained in the study of the 4H-SiC piezoresistive effect. Figure 7g shows the scheme of the radial arrangement of the piezoresistors, in which two groups of piezoresistors are located radially at the edge of the diaphragm and the other two groups are also radially arranged but located at the center of the circular diaphragm. With diaphragm deformation, the four groups of piezoresistors were subjected to parallel strain and current directions, and only the longitudinal piezoresistive effect of 4H-SiC was considered. In this case, when the piezoresistors are closer to the location of the maximum stress at the edge, there is a greater output sensitivity. Through the above systematic study, the arrangement scheme and location of the piezoresistors were optimized in detail to ensure high sensitivity of the 4H-SiC piezoresistive sensor chip in the design stage.

### Fabrication of the pressure sensor chip

During the fabrication of 4H-SiC piezoresistive pressure sensor chips, the most difficult processing consists of two aspects. First, because 4H-SiC is hard and does not react with acids and bases at room temperature, the wet etching method commonly used in Si processes is not applicable for 4H-SiC^49^, so the formation of a sensitive diaphragm uses mainly on dry plasma etching. The relevant research indicates that dry etching has the advantage of high quality, and by adding suitable oxygen, the products generated by etching can be removed in time to significantly enhance the rate. Second, as the connection point between chip signals and external devices, ohmic contacts are present in almost all sensor devices, including those with Si substrates. With its small forbidden bandwidth and suitable work function^50,51^, Si has proven able to develop linear current-voltage characteristics (ohmic characteristics) with conventional metals relatively easily. However, compared to Si, 4H-SiC as a wide band gap semiconductor has a large 4.95 eV work function^52^, which leads to a high Schottky barrier at the metal-SiC ohmic contact interface. Thus, the formation of current-voltage ohmic contact characteristics becomes a major challenge limiting a series of 4H-SiC electronics applications, including 4H-SiC pressure sensors. In addition, the metal combination used must be able to withstand high temperature conditions exceeding 500 °C, which makes the SiC ohmic contact an important challenge limiting the development of SiC devices. Based on this, a multilayer metal combination of Ni-based 4H-SiC ohmic contacts and Ta-based high-temperature resistant barrier layers has been used to form high-temperature SiC ohmic contacts and lead layers that overcome processing challenges.

**Fig. 8:**
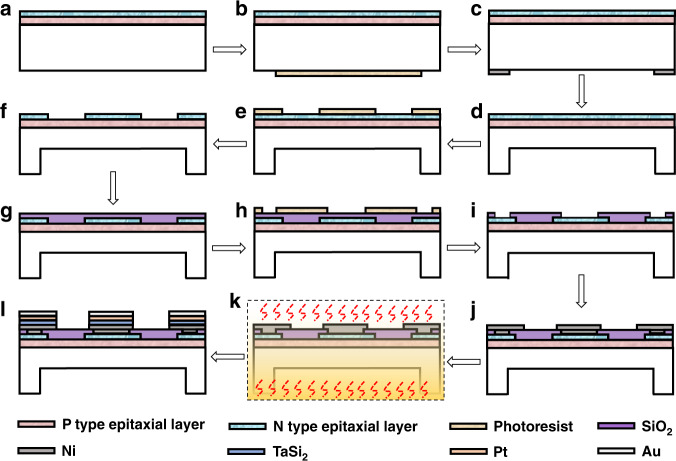
The complete process flow for chip fabrication. **a** Wafer cleaning using standard RCA procedures. **b** Lithographic patterning of sensitive diaphragm patterns. **c** Electroplating of masked Ni layer. **d** Deep dry etching of sensitive diaphragm back cavity. **e** Lithographic patterning of sensitive piezoresistors patterns. **f** Dry etching of sensitive piezoresistors. **g** PECVD of the SiO_2_ layer. **h** Lithographic patterning of ohmic contact areas. **i** Opening ohmic contact window. **j** Sputtering Ni metal layer and lift off. **k** Rapid thermal annealing. **l** Forming the metal lead layer

Figure 8 illustrates the complete process flow for sensor chip fabrication. (a) The 4H-SiC wafer was first immersed in a mixed solution of H_2_SO_4_ and H_2_O_2_ in a ratio of 2:1 at 100 °C for 10 min to remove organic impurities from the surface. Then, the diluted HF solution was used to remove oxides. After, an alkaline mixed solution of NH_4_OH: H_2_O_2_: H_2_O = 1:1:5 and an acidic solution of HCl: H_2_O_2_: H_2_O = 1:1:8 at 70-75 °C were used successively to remove the metal elements that might be present on the wafer surface. Finally, the wafer was rinsed several times with deionized water, making the wafer surface clean. (b) AZ4620 photoresist of 3 μm thickness was coated on the back side (C-side) of the 4H-SiC epitaxial wafers and photolithographically patterned to form a sensitive diaphragm pattern. The photolithography machine type was ABM6/350NAV13SV/M. (c) The Ni layer was grown by magnetron sputtering and plating in the area outside the patterned sensitive diaphragm. (d) Inductively coupled plasma etching (ICP) is used to form a sensitive diaphragm. The etching process parameters were 800 W RF power, CF_4_: O_2_ = 4:1 etching gas flow rate, 2400 W source power, and an etching rate of approximately 1200 nm/min. (e) AZ4620 photoresist of 8 μm thickness was coated on the n-type epitaxial layer (Si side) of the 4H-SiC epitaxial wafer and photolithographically patterned to form a piezoresistor pattern. (f) ICP shallow etching removes excess materials outside the n-type piezoresistor area to form a piezoresistor with a thickness of 2 μm. (g) A 300 nm thick SiO_2_ layer was deposited on the piezoresistor surface by ORION III type plasma-enhanced chemical vapor deposition (PECVD) for electrical insulation between the metal layers and substrate. (h) AZ4620 photoresist is again coated on the surface and patterned to form ohmic contact windows. (i) BOE solution was used to etch the excess SiO_2_ to open the ohmic contact window. (j) Ni (75 nm thickness) was magnetron sputtered on the front surface of the wafer using a DISCOVERY635-type magnetron sputtering machine. Then, ohmic contact electrodes were formed by the lift-off process. (k) 1000 °C, 120 s, N_2_ atmosphere high temperature Rapid Thermal Annealing (RTA) was conducted to form Ni/SiC electrical ohmic contacts, and the RTA furnace type is RTP-100 UniTemp. (l) The Ta-based multilayer metal combination TaSi_2_/Pt/Au was sputtered and patterned to form a metal lead layer to realize the electrical connection between the chip and the outside world and to cover and protect the ohmic contact layer. (m) UVCS-15X-type laser dicing is used to cut the wafer into individual unit sensor chips.

### Measurement setup

**Fig. 9:**
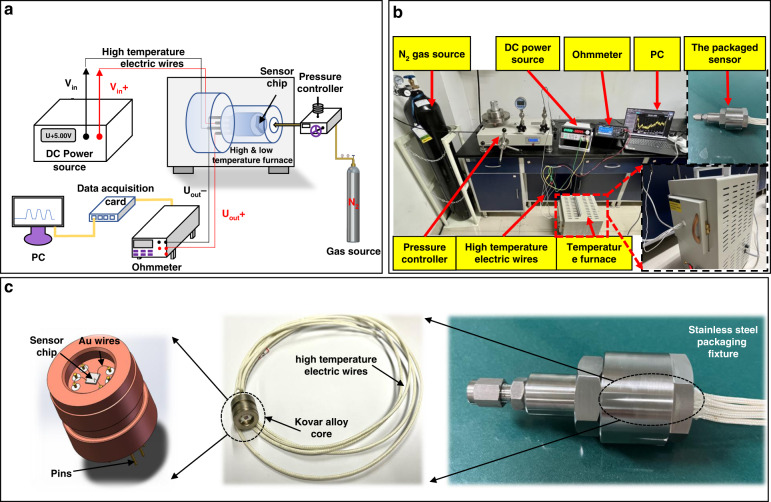
Experimental platform. **a** Schematic of the closed-loop test platform for the piezoresistive high-temperature pressure sensor. **b** Experimental system for the piezoresistive high-temperature pressure sensor. **c** High-temperature packaging details of the sensor chip

For experimentation on SiC high-temperature pressure sensor devices, in addition to the sensor chip itself, the construction of a static pressure calibration test platform is particularly important. Different from the Si-based pressure sensor test platform, the SiC high-temperature pressure sensor test platform faces more limitations. The most critical requirement is that all materials and equipment in the package and test, in addition to withstanding the pressure load of a specific range, must also be able to withstand the high temperature load. To solve this problem, a high-temperature pressure testing system with a pressure range of 6 MPa was built, as shown in Fig. 9a, b. The system consists of a PDQ-G6M-type piston barometer with a pressure range of 6 MPa, a 50 L nitrogen cylinder, a stainless steel pilot tube, a high-temperature furnace, a Keithley 34460 A ohm meter, and a Keithley 2400 source meter. Specifically, as shown in Fig. 9c, the piezoresistive SiC pressure sensor chip is encapsulated in a kovar alloy core, and then the whole is held in place by a metal stainless steel packaging fixture to create a sealed pressure space. The sensor is connected to the pressure controller through a pressure lead-in tube to form a pressure flow channel. For testing, the packaged sensor probe is placed in a high- and low-temperature furnace, the differential voltage signal from the sensor chip is output to an external ohmmeter via high-temperature electric wires, and the sensor Wheatstone bridge is powered by a DC 5 V voltage source.

## Supplementary information

SUPPLEMENTAL MATERIAL

## Data Availability

The data that support the findings of this study are available from the corresponding author upon reasonable request.
