# Supplementary material for: Exploring the nonlinear piezoresistive effect of 4H-SiC and developing MEMS pressure sensors for extreme environments
Source: Microsyst Nanoeng. 2023 Apr 3;9:41. doi: 10.1038/s41378-023-00496-1 (PMC10070454; doi:10.1038/s41378-023-00496-1)
Supplement: Supplementary file 1 — SUPPLEMENTAL MATERIAL [file 41378_2023_496_MOESM1_ESM.pdf]

Supplementary Material

Dear editor,

After carefully checking the editor and reviewers' comments and suggestions, we believe that all the comments are helpful and meaningful to improve the quality of this manuscript. Based on the comments, some supplementary materials have been added, which can make the methods be described in more detail, and provide reference for other related researchers.

- **Effect of substrate doping concentration on GF value and the doping concentration detection of 4H-SiC N type layer.**

First, according to the theory of solid physics, the doping process deviation may cause different GF values.

$$\begin{cases} GF = \dfrac{\Delta R}{R\varepsilon} \approx \dfrac{\Delta \rho}{\rho \varepsilon} \\ \rho = \dfrac{1}{\lambda} = \dfrac{1}{q\mu_e N_e} \end{cases} \tag{1}$$

In Eq. (1), ρ is resistivity, λ is the conductivity, μ_e is the electron carrier mobility, and N_e represents the doping concentration of N type layer. Therefore, according to the Eq. (1), doping concentration will affect the GF value of semiconductors.

Second, to avoid the difference in GF value at different positions on the original wafer caused by doping deviation, after completing the design of doping parameters, we used mature technology and high-end equipment to carry out the doped epitaxial growth process in a professional semiconductor wafer company, and tried our best to obtain lowest doping error. Figure 1 shows the secondary ion mass spectrometry (SIMS) analysis results of doping concentration in N-type layer of 4H-SiC epitaxial wafer. Along the depth direction,

the doping concentration fluctuates within an acceptable small range. Therefore, the uniform doping concentration obtained with the mature doping process will ensure the consistency of the resistivity at different positions, which avoids significant influence of the doping process on the material piezoresistive effect.

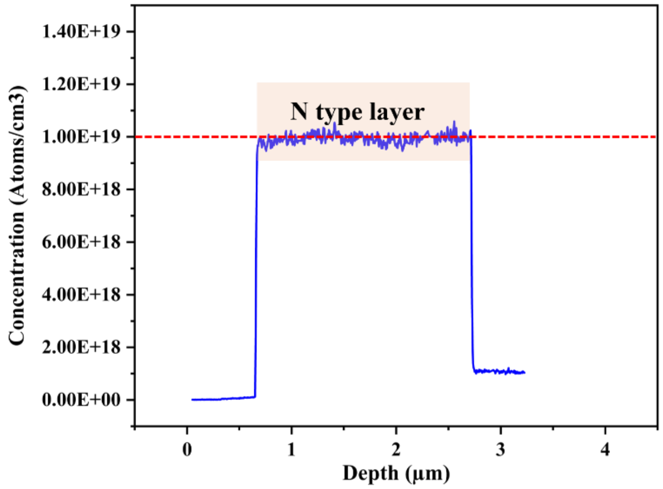

Figure 1. The secondary ion mass spectrometry (SIMS) analysis results of doping concentration in N-type layer of 4H-SiC epitaxial wafer.

- **In the study of piezoresistive effect, how to extract the strain value of the piezoresistors from the simulation results?**

When studying the piezoresistive effect of materials, the beam bending method has the advantages of simple structure and convenient to calculation. It is also applied in the research of other amorphous materials. As shown in Figure 2, the piezoresistive effect is studied using the cantilever beam bending method. Specifically, in the experiment, we prepared a cantilever beam with a size of 33 mm × 5 mm × 0.35 mm, and then fabricated piezoresistors on the surface at the distance of 11.45 mm from the fixed end by MEMS technology. Then, a piezoresistive cantilever beam test platform was built as shown in

Figure 2a. One end of the cantilever beam was fixed, and a specific force F was applied to the other end. At this time, the cantilever beam was bended, and the piezoresistors on it would generate stress and strain.

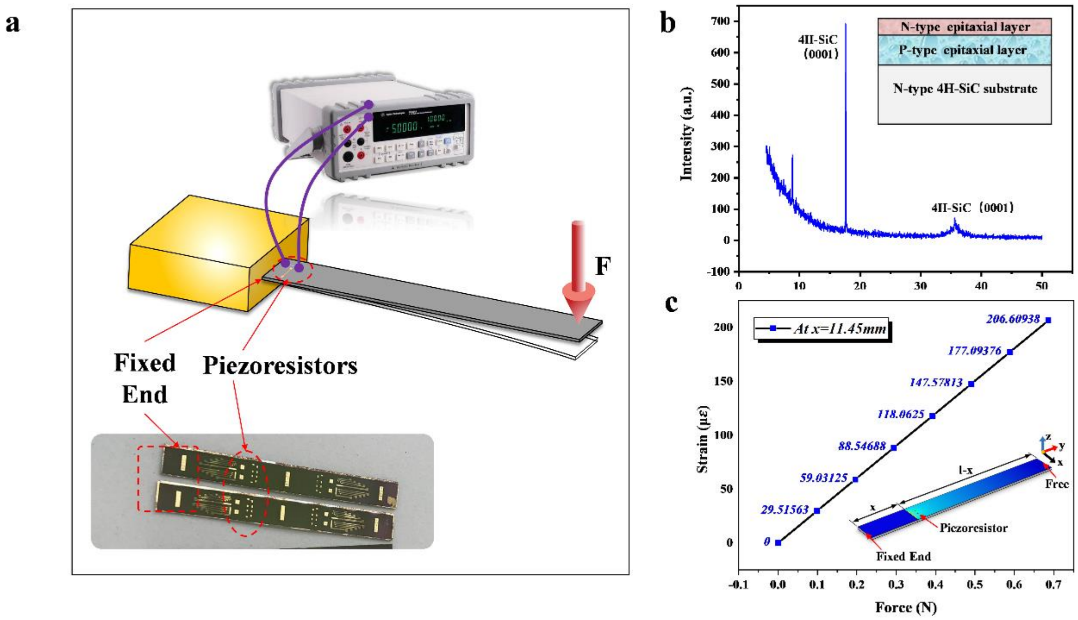

Figure 2. Piezoresistive effect of N-type 4H-SiC. **a** Schematic diagram of the experimental setup for piezoresistive effect testing. **b** 4H-SiC epitaxial wafer parameters and its X-ray diffraction characteristic peaks. **c** Surface strain distribution of piezoresistive test samples under F load.

To further calculate the piezoresistive coefficient GF, the strain value of the piezoresistors on the cantilever beam need to be determined by simulation. Specifically, a cantilever piezoresistive model was established as shown in Figure 3. The sample size and piezoresistors distribution in the model are consistent with the experimental sample, as shown in the embedded rectangular cantilever structure in Figure 2a. With reference to the experiment, the same loads and constraints were imposed on the cantilever in the software (One end was fixed, and force F was applied at the other end), then the strain induced in

the piezoresistors were extracted, which is summarized in the $F \sim \varepsilon$ curve as shown in Figure 2c.

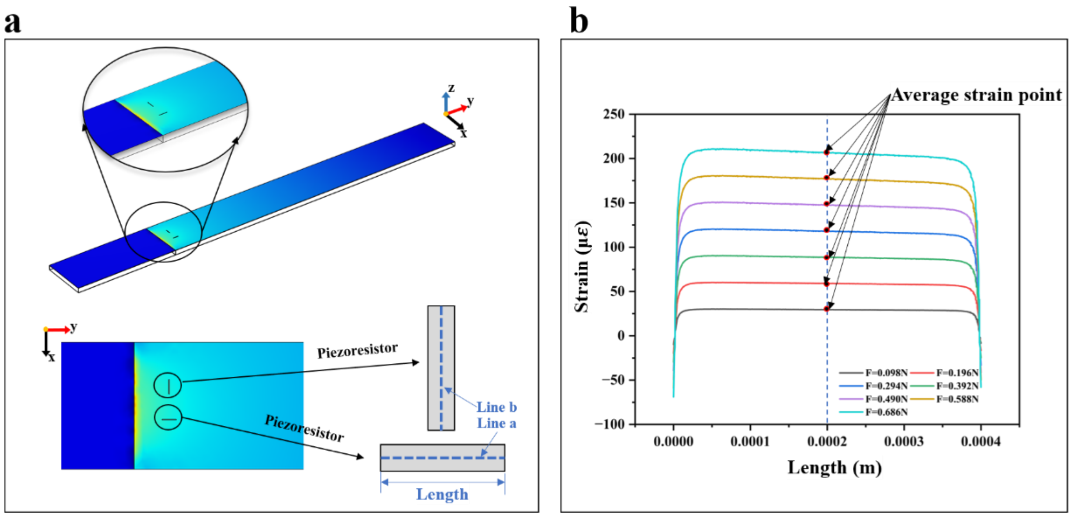

Figure 3. **a** Cantilever model for the piezoresistors. **b** Strain distribution along piezoresistors' length direction and the equivalent extraction of average strain value.

As shown in the Figure 3b, we displayed the strain distribution along piezoresistors' length direction, the strain of the piezoresistors in the layout direction is a partial strain. Although the size of the piezoresistors are quite small, the strain value along the length and width of the piezoresistors change with locations. When calculating GF value, a specific strain value of the piezoresistor under each different load *F* need to be extracted from the simulation results. **Considering the linear distribution of strain values on the piezoresistors, we take the strain value at the geometric center point of the piezoresistor as the final average strain value. As shown in the Figure 3a, after the simulation, first, define a line "a" along the length direction at the width center point of the piezoresistor. And then, draw the strain value along the line "a" as shown in Figure 3b. Next, extract the strain value at the center point along the length direction. Through the above method, we extracted the strain value at the geometric center of**

the piezoresistor, which is also equal to the average strain value of the whole piezoresistor.
